# Assessing the Physiological Effects of Traditional Regional Diets Targeting the Prevention of Cardiovascular Disease: A Systematic Review of Randomized Controlled Trials Implementing Mediterranean, New Nordic, Japanese, Atlantic, Persian and Mexican Dietary Interventions

**DOI:** 10.3390/nu13093034

**Published:** 2021-08-30

**Authors:** Markos Klonizakis, Alex Bugg, Beatrice Hunt, Xenophon Theodoridis, Dimitrios P. Bogdanos, Maria G. Grammatikopoulou

**Affiliations:** 1 Lifestyle, Exercise and Nutrition Improvement (LENI) Research Group, Sheffield Hallam University, Sheffield S10 2BP, UK; a.bugg@shu.ac.uk (A.B.); beatriceehunt@gmail.com (B.H.); 2 Department of Nursing and Midwifery, College of Health, Wellbeing and Life Sciences, Sheffield Hallam University, Sheffield S10 2BP, UK; 3 Medical School, Faculty of Health Sciences, Aristotle University of Thessaloniki, University Campus, 54124 Thessaloniki, Greece; xenophontheodoridis@gmail.com; 4 Department of Rheumatology and Clinical Immunology, Faculty of Medicine, School of Health Sciences, University of Thessaly, Biopolis, 41334 Larissa, Greece; bogdanos@med.uth.gr (D.P.B.); maria@ihu.gr (M.G.G.); 5 Department of Nutritional Sciences & Dietetics, Faculty of Health Sciences, Alexander Campus, International Hellenic University, 57400 Thessaloniki, Greece

**Keywords:** NCD, obesity, hypertension, medical nutrition therapy, nutrition transition, territorial diet, noncommunicable disease, cardiovascular health, inflammation, sustainable diet

## Abstract

Traditional regional diets are considered as sustainable dietary patterns, while many have been examined with regard to their health benefits. The aim of the present systematic review was to aggerate all evidence on the physiological effects of regional diets among adults at high risk for cardiovascular disease (CVD). Three databases were searched for randomized controlled trials (RCTs) implementing any regional diet (Mediterranean (MedD), Persian, Southern European Atlantic, Japanese, Chinese, new Nordic, or other) while examining cardiovascular risk factors among adults at increased risk. Primary outcomes included anthropometric indices and secondary outcomes involved blood lipid concentrations, glucose metabolism, inflammation and other markers of CVD progression. Twenty RCTs fulfilled the study’s criteria and were included in the qualitative synthesis, with the majority implementing a MedD. Adherence to most of the regional diets induced a reduction in the BW and anthropometric indices of the participants. The majority of RCTs with blood pressure endpoints failed to note a significant reduction in the intervention compared to the comparator arm, with the exception of some new Nordic and MedD ones. Despite the interventions, inflammation markers remained unchanged except for CRP, which was reduced in the intervention groups of one new Nordic, the older Japanese, and the Atlantic diet RCTs. With regard to blood lipids, regional diet interventions either failed to induce significant differences or improved selective blood lipid markers of the participants adhering to the experimental regional diet arms. Finally, in the majority of RCTs glucose metabolism failed to improve. The body of evidence examining the effect of regional dietary patterns on CVD risk among high-risk populations, while employing an RCT design, appears to be limited, with the exception of the MedD. More research is required to advocate for the efficacy of most regional diets with regard to CVD.

## 1. Introduction

Noncommunicable diseases (NCDs), including ischemic heart disease, stroke, cancer, diabetes and chronic lung disease, are collectively responsible for almost 71% of global deaths—over 41 million in total [1]. Among these, 15 million are considered as “premature” deaths, occurring between the ages of 30 and 69 years old [1] and could potentially be avoided. NCDs come also with a large financial burden to both the society, the individual household level and the national healthcare systems [2,3] as well as a substantial disability burden [4].

**Figure 1:**
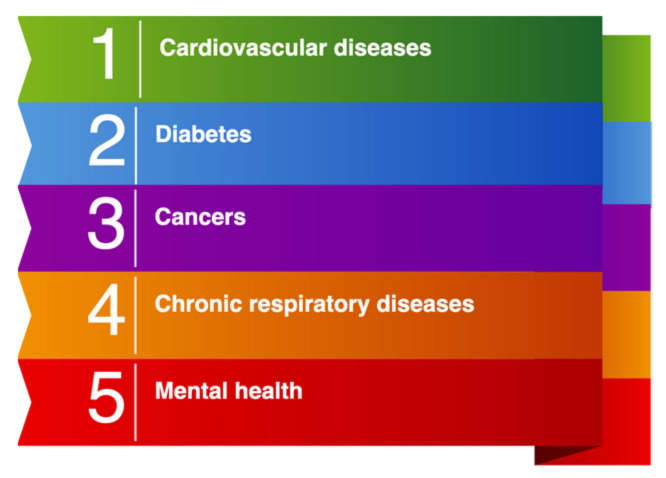
Non-communicable diseases categories.

NCDs are the confluence of genetics, physiological, behavioral and environmental triggers [5]. As per the World Health Organization (WHO), the NCDs include five main categories namely cardiovascular diseases (CVDs), cancers, chronic respiratory diseases, diabetes [5] (Figure 1), and the more recent inclusion of mental health and wellbeing [6]. These five categories often co-occur, sharing intertwined causes. Proximal causes for the development of NCDs include elevated total cholesterol (TC), blood pressure (BP), and fasting plasma glucose (FPG) levels, whereas intermediate causes involve tobacco smoking, unhealthy diets, physical inactivity and excessive alcohol consumption [7].

Among all NCDs, CVDs consist of the leading cause of disease burden globally, demonstrating an alarmingly high rate [8]. Moreover, over the past few years, the global epidemiological CVD landscape has changed dramatically, with unhealthy diet becoming an increasingly important modifiable factor for the development of CVD causes [9].

Nutrition-based lifestyle interventions have been recommended as a primary and secondary prevention strategy for NCDs and for CVD in particular, for all high-risk populations (e.g., those who are obese, physically inactive, smoking, consuming unhealthy diets or excessive amounts of alcohol, etc., [10,11,12]). According to the WHO, globalization has produced a global deviation in dietary patterns, known as nutrition transition [13,14,15], which is responsible for the recorded cumulative increase in NCD burden, regarding the CVD domain in particular [11]. For this, the effect of traditional regional diets (i.e., traditional dietary patterns from different regions of the world) have been extensively examined for the prevention of NCDs. For example, Mediterranean diet (MedD) interventions have been suggested to reduce CVD risk among older adults or post-menopausal populations, both as standalone dietary interventions [16,17] or in conjunction with exercise [18,19], offering both short- [17,18,19] and long-term benefits [20]. Similar findings have been reported in studies assessing the new Nordic [21,22], the Japanese [23], the Southern European Atlantic [24], and the Mexican [25] regional diets.

The aim of the present systematic review was to aggerate all available evidence on the physiologic effects of regional diets among adults at high risk for developing CVD.

## 2. Materials and Methods

### 2.1. Research Question, PICO and Study Protocol

The research question was “what is the effect of regional diets in ameliorating anthropometric, biochemical, and cardiovascular indices in adults at high risk for developing CVD?” Randomised controlled trials (RCTs) were screened according to the population, intervention, comparison, and outcome (PICO) criteria (Table 1).

The protocol was registered at PROSPERO (CRD42020201200), and the review is presented in accordance with the Preferred Reporting Items for Systematic Reviews and Meta-Analysis (PRISMA) guidelines.

### 2.2. Search Strategy

The PubMed, Excerpta Medica Database (Embase) and Cochrane Central Register of Controlled Trials (CENTRAL) databases were searched from inception until August 2020. In parallel, the search was also extended to the gray literature. Used search terms, with a combination of MeSH terms whenever applicable in each database, included: (Japanese diet), (Mediterranean diet), (Nordic diet), (Atlantic diet), (Mexican diet), (Persian diet), (Chinese diet), (obesity), (adiposity), (adipose tissue), (body fat), (overweight), (obese), (body mass index), (blood pressure), (hypertension), (hypertensive), (insulin), (insulin sensitivity), (diabetes), (diabetic), (hyperlipidaemia), (cholesterol), (triglyceride), or (weight). A detailed search string for the PubMed database is presented in Table 2.

### 2.3. Inclusion and Exclusion Criteria

Inclusion criteria for the synthesis involved (1) publications written in the English language, (2) with a RCT design (parallel or cross-over), (3) with participants having a good general health but who were high risk for the development of CVD according to the WHO (age ≥55 years old, and at least one risk factor including post-menopause, hyperglycemia or prediabetes, hypertension, hyperlipidaemia, overweight or obesity, cigarette smokers, excessive alcohol drinkers, or physically inactive), (4) with one intervention implementing a traditional regional diet only, (5) a comparator arm implementing any other diet, sham diet, or no intervention, and (6) the duration of intervention lasting for at least 4 weeks.

Exclusion criteria involved (1) publications written in languages other than the English, (2) lacking an RCT design, (3) with participants having a confirmed diagnosis of chronic obstructive pulmonary disease, Parkinson’s Disease, any form of cancer, or diabetes mellitus diagnosis, (4) incomplete trials, (5) lacking an intervention implementing a traditional territorial diet, (6) implementing a parallel exercise intervention, (7) involving younger participants.

### 2.4. Outcomes of Interest

Primary outcomes included body weight (BW), body mass index (BMI), waist circumference (WC), waist-to-hip ratio and abdominal adiposity.

Secondary outcomes included body fat (as a % of BW), systolic blood pressure (SBP), diastolic blood pressure (DBP), FPG, fasting insulin (Ins), insulin resistance (IR), glycosylated haemoglobin (HbA1c), TC, low-density lipoprotein (LDL), high-density lipoprotein (HDL), TC:HDL ratio, and Flow Mediated Dilatation (FMD), as well as inflammation and atheromatosis markers.

### 2.5. Data Extraction

An a priori data extraction form was created in Microsoft Excel (version 16.0). Two researchers (A.B. and B.H.) independently extracted all of the data using the form, with two senior researchers (M.K. and M.G.G.) independently checking all of the data for consistency.

Extracted data included bibliographic information, study, and participant characteristics, randomization, wash-out periods (in case of cross-over studies), intervention characteristics, treatment duration, and comparator and outcome data including any relevant parameters named in the primary and secondary outcomes. In the case of any missing or unclear data, two attempts were made to contact the corresponding author by email. If no response was received, the missing data were not included.

### 2.6. Risk of Bias in Individual Studies

The Cochrane Risk of Bias (RoB) 2.0 tool [26] was used to assess the quality at the study level as having a high, low, or unclear risk of bias. The tool evaluates studies based on seven criteria: (1) randomization generation, (2) allocation concealment, (3) blinding of outcome assessors, (4) blinding patients/study personnel, (5) incomplete outcome data (that is, lost to follow-up), (6) selective outcome reporting, and (7) other risks of bias.

## 3. Results

### 3.1. Search Results

**Figure 2:**
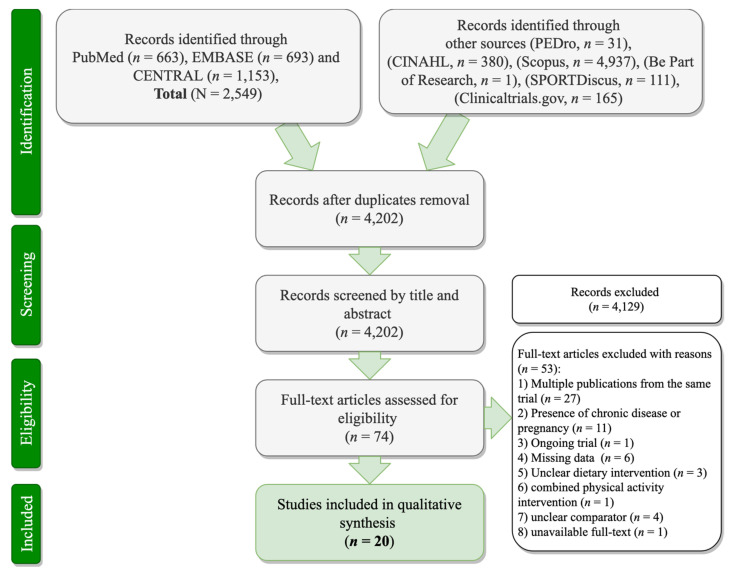
PRISMA flowchart of the study selection process.

The initial search of databases identified a combined total of 8173 records. After screening the titles, a total of 20 distinct RCTs fulfilled the predefined criteria and were subsequently included in the qualitative synthesis. A detailed PRISMA flow chart of the RCT selection process is presented in Figure 2.

### 3.2. Characteristics of the Included RCTs

#### 3.2.1. Intervention and Comparator Arms

Table 3 describes the characteristics of the included RCTs. One RCT assessed the effect of the traditional Japanese diet [27,28], another implemented a traditional Chinese diet intervention [29], three RCTs evaluated a new Nordic diet intervention [30,31,32], two RCTs assessed the effect of the Traditional Persian Medicine (TPM) diet [33,34], one trial evaluated adherence to the Mexican diet (MexD) [35], one the Southern European Atlantic Diet (SEAD) [24] and a total of 12 RCTs assessed the results of MedD interventions [36,37,38,39,40,41,42,43,44,45,46,47,48,49].

Traditional territorial diets were compared against average modern diets of the same region [27,28,30,32,36,37], the typical western/US diet [29,31,35,47], the habitual diet of inhabitants of that area [24,38,39,40,41,48,49], known healthy diets such as the low-fat diet [33,39,42,43]; the dietary approaches to stop hypertension (DASH) diet [45]; hypocaloric diets with oral nutrient supplements (ONS) [34]; diets supplemented with fish oil, walnuts, and grape juice [38]; weight-loss medication [33,34]; the Atkins diet [46]; or a lacto-ovo vegetarian diet [44].

#### 3.2.2. RCT Design, Masking and Duration

Most RCTs were of parallel design, with a small number (*n* = 4) implementing cross-over interventions [35,42,43,44]. There were three trials that had double-blind masking [32,45], four were single-blind [34,35,36,37,39], the majority were open label [24,30,33,38,40,42,44,46,47,48,49,50], and one did not report blinding at all [29]. Intervention duration ranged between 28 days [27,28] and a total of 26 weeks [30].

#### 3.2.3. RCT Population

The pooled number of participants reached a total of 3486 participants. Most studies used patients with overweight simple or abdominal obesity [27,29,30,34,35,38,45,46,47], patients with hypercho-lesterolaemia [31,32,42,43] or at least one cardiometabolic risk factor [42,43,44], elevated hs-CRP [32], NAFLD [33], or older adults [40,48,49].

#### 3.2.4. Outcomes of the Included Trials

Outcomes of cardiovascular (CV) interest in the included RCTs involved anthropometric indices (BW, BMI, waist circumference, hips circumference, BF, FFM, VAT, hips-to-waist ratio), indicators of glucose and insulin metabolism (FPG, Ins, HOMA-IR, Matsuda Index, HbA1c), changes in the NAFLD grade, blood pressure (SAP and DAP), FMD, inflammation markers (TNF1, IL-6, CRP, etc.), indirect carotid intima-media thickness via asymmetric dimethylarginine (ADMA) assay, blood lipid concentrations (HDL, LDL, TC, TG, apolipoproteins, etc.) and cytokines levels.

Moreover, few trials also included non-CV-specific outcomes, like stress, cognitive function, well-being, depression [39], 8-iso-prostaglandin F2a (8-iso-PGF2a), a marker of oxidative stress [46], sagittal diameter, physical fitness [30], blood mineral concentrations [27,28], hunger, as well as subjective physical and mental health [29].

### 3.3. Characteristics of the Traditional Regional Diets

**Figure 3:**
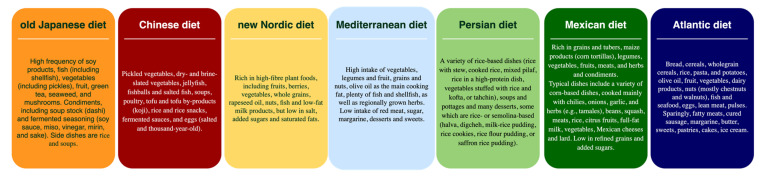
Characteristics of the regional diets included in the systematic review.

Characteristics of the traditional regional diets included in the RCTs are presented in Figure 3. The traditional Japanese diet is based on several elements, including a high consumption of soy products, fish and shellfish, vegetables (pickles in particular), seaweed, mushrooms, fruit, and green tea [27,51]. Specific condiments of the dietary pattern include dashi (soup stock) and fermented seasoning (soy sauce, miso, vinegar, mirin, and sake), with rice and soup being consumed as part of the meals.

The traditional Chinese diet is similar to the Japanese one, including pickled vegetables, dry- and brine-slated vegetables, jellyfish, fish-balls and salted fish, soups, poultry, tofu and tofu by-products (koji), rice and rice snacks, fermented sauces, and eggs (salted and thousand-year-old) [52].

The new Nordic diet [53] is rich in high-fiber plant foods (fruits, berries, vegetables, whole grains, nuts), rapeseed oil, fish, and low-fat milk products. Salt intake, sugar, and saturated fats are rarely consumed.

Characteristics of the MedD include a high intake of vegetables, legumes and fruit, grains and nuts, olive oil as the main cooking fat, plenty of fish and shellfish as well as regionally grown herbs [54,55,56].

The traditional Persian diet includes a great variety of rice-based dishes (rice with stew, cooked rice, mixed pilaf, rice in a high-protein dish, vegetables stuffed with rice and kofta, or tahchin), soups and pottages, and many desserts, some which are rice- or semolina-based (halva, digcheh, milk-rice pudding, rice cookies, rice flour pudding, or saffron rice pudding) [57].

As for the traditional Mexican diet, it is rich in grains and tubers, maize products (corn tortillas), legumes, vegetables, fruits, meats, herbs and condiments [25]. Typical dishes include a variety of corn-based dishes that are mainly cooked with chilies, onions, garlic, and herbs (e.g., tamales), beans, squash, meats, rice, citrus fruits, full-fat milk, vegetables, Mexican cheeses, and lard [35]. Moreover, the diet is low in refined grains and added sugars [58].

Finally, the Atlantic diet is based on a frequent consumption of bread, cereals (including whole-grain), rice, pasta, potatoes, fruit, vegetables (including vegetable soup) and olive oil, a daily intake of dairy products, frequent consumption of nuts (preferably chestnuts or walnuts), fish (in particular cod) and seafood [59,60]. Eggs, lean meat, and pulses are also consumed on a weekly basis (2–3 times), whereas fatty meats and cured sausages, butter and margarine as well as desserts, sweets, pastries, cakes, and ice cream are sparingly consumed [60].

### 3.4. Effects of Regional Diets on CVD Prevention among Participants with Increased CVD Risk

Table 4 details the CVD outcomes of the individual RCTs favoring the regional diet intervention arms.

Adherence to most of the regional diets induced a reduction in the BW and anthropometric measures of participants.

The majority of trials with BP endpoints failed to note a significant reduction in the intervention compared to the comparator arm, with the exception of some new Nordic [30,31] and MedD [40,43,48,49] interventions.

Despite the interventions, inflammation markers remained unchanged, except for CRP, which was reduced in the intervention groups of one new Nordic [25], the older Japanese [27,28], and the Atlantic diet [24] RCTs.

With regard to blood lipid concentrations, the regional diet interventions either failed to induce significant differences or improved the lipid profile of participants adhering to the experimental regional diets.

Finally, in the majority of the RCTs, glucose metabolism failed to improve in participants with increased CV risk.

#### 3.4.1. Japanese Diet and CVD Prevention

One trial [27,28] assessed the effect of the older Japanese diet compared against the modern Japanese diet among patients with overweight/obesity. Anthropometric indices (BW, BMI and body fat mass) were improved in the arm adhering to the older Japanese diet, whereas in parallel, HDL, LDL, CRP and HbA1c were also improved. No changes were noted with regard to the FPG, IR evaluated through the homeostatic model assessment for IR (HOMA-IR), alanine transaminase (ALT), or blood pressure.

#### 3.4.2. Chinese Diet and CVD Prevention

Leonetti and associates [29] compared the Chinese diet against the typical Western one, both in a hypo-caloric version, among patients with overweight/obesity. After six weeks of intervention, BMI, lean body mass (LBM), subjective hunger, as well as reported physical and mental health indicators were improved in the arm adhering to the Chinese diet.

#### 3.4.3. New Nordic Diet and CVD Prevention

Among the three RCTs evaluating a new Nordic diet intervention (NorD) [30,31,32], one used the typical Western diet as a comparator [31], and the remaining assessed differences between the new Nordic diet (a healthier version of the diet) compared to the typical/average contemporary Danish diet [30,32].

Adherence to the new Nordic diet for 6 weeks [31] among patients with hypercholesterolaemia induced a significant reduction in the BW and BMI of the participants in the intervention arm compared to those allocated to the Western diet group. SBP, fasting insulin, TC, LDL, HDL, LDL/HDL ratio, apolipoprotein A_1_ (ApoA_1_) and B (ApoB) concentrations as well as their ratio (apoB/apoA_1_) were also improved. No differences were recorded with regard to the FPG and DBP levels.

When the new Nordic diet was compared against the typical Danish diet among patients with abdominal obesity [30], all of the anthropometric indices were improved in parallel to the SBP, IR, TG, TC, and CRP levels. However, no differences were noted in the FPG and insulin levels between treatment arms. Adhering to a high-fiber Nordic diet for eight weeks [32] improved BW and blood lipid levels (TC TG, LDL and HDL) among participants with elevated TC and high-sensitivity CRP (hs-CRP) at baseline. The intervention failed to induce improvements with regard to blood pressure, FPG, insulin, HbA1c, hs-CRP, interleukin-6 (IL-6), ALT, interferon γ (IFNγ), soluble tumor necrosis factor receptor 1 (sTNFR1), apoB, or apoA_1_ levels_._

#### 3.4.4. Mediterranean Diet and CVD Prevention

Compared to a Western-style diet, the MedD was ineffective in improving the anthropometric, lipid, and FPG profile of non-diabetic patients with abdominal obesity [47] after 10 weeks of intervention. On the other hand, when participants with overweight or simple obesity were recruited [38], BW, TC, and LDL were significantly improved within 8 weeks in the treatment arm compared to the group retaining their usual diet alone or supplemented with fish oil, walnuts, and grape juice.

When the comparative effectiveness of an isocaloric MedD was compared against the DASH diet for 12 weeks [45] in a similar sample, all of the athropometric indices (BW, WC, BF, and visceral adipose tissue [VAT]) and additionally ALT were improved in the MedD arm. In a 2-month comparative effectiveness trial where one group of women with overweight/obesity adhered to a hypo-caloric MedD and another to the Atkins diet [46], BW and FMD were significantly improved in the MedD arm. When the MedD was compared against an energy-restricted Central European dietary pattern for 6 weeks, BW, BF, FFM, and VAT were all reduced among post-menopausal women with overweight/obesity [36,37]. Nevertheless, no differences were recorded in the ADMA or hs-CRP concentrations of participants.

The Cardiovascular Prevention with Vegetarian Diet (CARDIVEG) [44] trial assessed the effect of a hypo-caloric MedD compared against a low-calorie lacto-ovo vegetarian diet for 3 months, in patients at increased CVD risk. Among a variety of hematological and anthropometric outcomes, only LDL and TG levels were improved.

When older adults were used as a sample, a yearly MedD intervention was effective in improving SBP [40,48,49]. Moreover, the Mediterranean diet for cognitive and cardiovascular health in the elderly (MedLey) trial [48,49] revealed additional improvements regarding the DBP, FMD, TG, and F2-isoprostanes levels at 6 months of intervention.

In the MedDairy [43] and MedPork [42] trials, Wade and associates compared the MedD supplemented with servings dairy or lean pork, respectively, against a low-fat diet for 8 weeks among patients at risk for CVD. In MedDairy [43], reductions in the anthropometric indices were noted (BW and BF), paired with improved SBP, HDL, and TG levels. In MedPork [42], the BW, BMI, and WC of the participants were improved. None of trials managed to improve LDL, DBP, FPG, fasting insulin, or TC levels.

When patients with mildly/moderately elevated TC levels were recruited, adherence to the MedD induced significant improvements in the BW, TC, and LDL levels compared to either a low-fat diet or the usual diet of the participants [39]. Nonetheless, measures of stress, cognitive function, well-being, and depression failed to change despite the intervention.

#### 3.4.5. Mexican Diet and CVD Prevention

Santiago-Torres and colleagues [35] evaluated a traditional Mexican diet intervention compared against the US diet in women with overweight/obesity for a total of 24 days in a cross-sectional manner. Among the plethora of evaluated outcomes only fasting insulin levels were improved post-intervention, with FPG, CRP, insulin-growth factor 1 (IGF-1), insulin-like growth factor binding protein 3 (IGFBP-3), adiponectin, IL-6, and IR failing to be improved.

#### 3.4.6. Traditional Persian Medicine Diet and CVD Prevention

Two RCTs compared the TPM against hypo-caloric diets [33,34] in patients with NAFLD [33] and women who were overweight [34], respectively. In the first RCY [33], BMI, NAFLD grade, ALT, and aspartate aminotransferase (AST) levels were significantly improved in the TPM arm, as compared to the low-fat hypo-caloric diet group. In the second trial, no improvements were noted, either when compared against the typical hypo-caloric diet (1200–1600 kcal/day) plus orlistat, or when compared to the typical hypo-caloric diet plus Majoon Davaye Balgham (MDB) ONS.

#### 3.4.7. Traditional Southern European Atlantic Diet and CVD Prevention

The GALIAT study [24] assessed the results of a traditional Atlantic diet intervention in in 250 families (irrespective of their health status), compared to their habitual diet. The intervention induced a reduction in anthropometric indices (BW, BMI, BF), as well as in the TC and LDL concentrations. No differences were reported with regard to inflammation markers.

### 3.5. Risk of Bias

**Figure 4:**
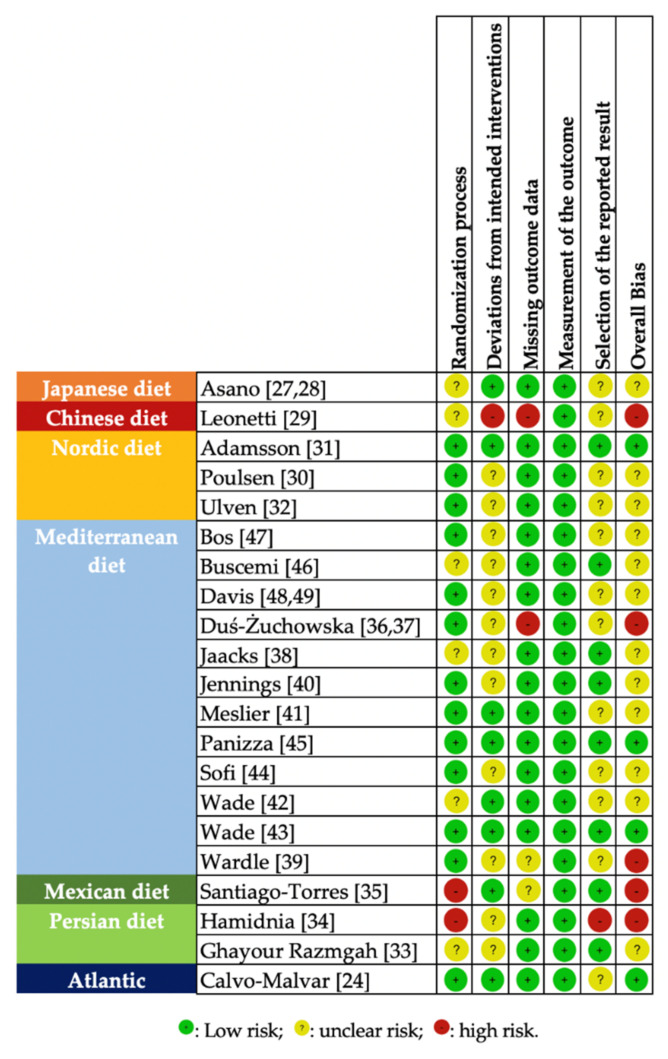
Randomized controlled trials, investigating the effects of MD interventions, rated by the Cochrane risk of bias tool [26].

A summary risk of the bias of the included RCTs is presented in Figure 4. The majority of RCTs had an unclear overall risk of bias, three had a low overall bias, and five were classified as having a high overall risk of bias.

### 3.6. Synthesis of the Outcomes

Although the number of studies and the total patient population were adequate, the high clinical and methodological heterogeneity did not allow for a synthesis of the outcomes.

## 4. Discussion

The present study revealed that research applying regional dietary interventions while examining CVD outcomes is very limited. From the examined dietary patterns, the MedD appears to be the most researched diet using an RCT design. Although individual RCTs indicate selective improvements in CVD outcomes following adherence to regional dietary interventions, any extrapolation of the findings for the formulation of recommendations would be rather premature and hasty.

### 4.1. Do Health Benefits Lie in the Constituents/Nutrients of Regional Diets?

**Figure 5:**
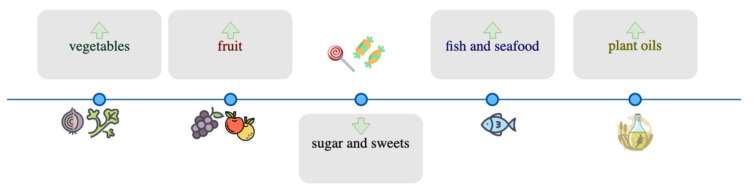
Shared food-group components of regional diets included in the present analysis.

According to Cena and Calder [61], healthy dietary patterns are apparent in certain regions of the world and are rooted in the local tradition while making use of the naturally available food sources in a sustainable manner [62]. Thus, to evaluate the potential beneficial effects of regional diets on CVD risk factors, it seems appropriate to consider the food sources, and quantities thereof, provided in the pre-scribed dietary interventions that might consist of the driving forces behind the improved health outcomes. Common denominators in the healthier, regional dietary interventions include a high fruit and vegetable intake, the consumption of fish and plant oils as well as the low intake of fat, sugar, sweets, and desserts (Figure 5). For each one of these diet components, a great body of evidence is backing the health benefits associated with its consumption.

Collectively, the dietary interventions implemented herein suggest an increased consumption of fruits and vegetables. This pattern has been shown to reduce serum TC in healthy men and women [6] and has been inversely related to LDL concentrations in healthy individuals and individuals with T2DM or coronary artery disease (CAD) [63]. Fruits and vegetables have a high fiber content, which, in turn, may reduce the glycemic index of foods, conferring benefits against CVD or its precursors (glycemic control, BW, etc.) [64].

Moreover, a high fruit and vegetables intake usually coincides with a reduced intake of sweets and sugar, indicating the adoption of a “healthier” dietary pattern. Literature is unanimous on the beneficial effects of low sugar intake on CV health [65,66], with benefits spanning from low subclinical inflammation [67], improved gut microbiota [68], and the attainment of a “healthy” body weight [69].

With regard to the fat intake, several studies have associated a higher fats intake, particularly SFA, to greater LDL concentrations, and the development of inflammation, T2DM and dyslipidemia [70]. Indeed, for many years, even the smallest intake of fat was “demonized” and a high-fat diet was a synonym to a higher CVD-risk diet [71]. Recently however, evidence from studies of higher hierarchy, including dose-response meta-analyses, has contradicted this narrative, suggesting that SFA intake has either a null effect, or even a positive one on the CV health according to the results of dose-response analyses [71,72,73]. Moreover, according to a recent Cochrane systematic review [74], cutting down on SFA for a period of four years has little or no effect on non-fatal myocardial infarction, coronary heart disease (CHD) and cancer mortality/incidence, diabetes diagnosis, HDL and serum TG concentrations, or blood pressure, and unclear effects on total myocardial infarction (MI), stroke and CHD events. Moreover, the number-needed-to-treat (NNT) for an additional beneficial outcome based on the primary prevention trials was 56, translating to 56 people needed to reduce their SFA intake for four years, in order for one person to avoid a CVD event [74]. When secondary prevention trials were accounted for, the NNT was calculated at 32 [74]. Accordingly, some researchers claim that the absolute intake of macronutrients appears to be less relevant to health than the selected food sources [75].

A high fish intake is an indisputable component of most of the regional diets included herein, and interestingly, all regional diets included in the present analysis involved countries with direct access to the sea. Research has showed that fishing is an important contributor to food security in water surrounding regions [76], and seafoods form both a traditional component of the regional diets, as well as an important effector of diet quality [77]. Moreover, according to meta-analyses of observational studies, greater fish intake is associated with a lower CHD incidence and mortality [78], as well as stroke risk [79].

Despite the health effects demonstrated by the consumption of individual foods and food-groups, as Schoenfeld and Ioannides promptly noted, it is risky to associate individual foods and nutrients with distinct health outcomes, as nutritional epidemiology is often biased by its limited reproducibility [54,80].

### 4.2. Concerns Regarding the Methodology of the Included RCTs

In the Japanese diet trials, the intervention arm (traditional diet) contained an increased quantity of soy products, seafood, fruits and vegetables, seaweed, and mushrooms compared to the comparator [27]. The 1975 Japanese diet servings contained approximately 4 g less fat per 100 g, 1 g more protein and 1 g extra carbohydrate compared to the modern diet. Differences in the macronutrient content of the two arms appear neglectable or non-different between arms; thus, it is the actual foods and their constituents that may have propelled the changes in the metabolic profile of participants, although this is difficult to discern when only one RCT was included.

In the Chinese diet RCT, the reported dietary information of the two arms was poor. Although there were positive significant outcomes reported for the intervention group, the precise nutritional content of the intervention/control diet that was consumed by the participants was not reported, so potential mechanisms could not be explored. Both diets provided approximately 1200 kcal/day, with participants given a variety of different food choices per meal, with set portion sizes. Caloric restriction, whereby the calorie intake is well below what would be consumed ad libitum (>10%), has been shown to have a positive effect on CV health [81]. The effects of caloric restriction in humans include beneficial changes in blood pressure, lipid profile, CV function and inflammatory response [82,83,84]. It seems possible that a contributing factor towards the positive changes in the Chinese diet intervention arm was the result of the caloric restriction. Moreover, since the comparator arm was also energy restricted, the reported results might be attributed to the specific nutrients in the prescribed diets. However, without knowing which nutrients were consumed by the participants, it is difficult to draw conclusions.

Three studies investigating the Nordic diet were included in this review. The new Nordic diet was shown to have positive effects on anthropometric indices and the lipid profile among obese participants and individuals with elevated TC, respectively [30,32]. The key features of the new Nordic diet, as reported by the authors, include replacing the SFA content of the diet with MUFA and PUFA sources, reducing the consumption of processed and refined grains, and increasing the intake of organically grown fruits and vegetables. In Ulven’s study, the SFA content was 19.2 g/day in the control arm and as low as 5.7 g/day in the intervention group [32]. The respective PUFA content reached 5.1 g/day and 14.4 g/day in the control diet and intervention diet, respectively. With regard to the fat content, as previously mentioned, recent research of a higher hierarchy disputes the efficacy of SFA replacement in improving CV risk [74]. Thus, the induced changes in the new Nordic diet arms rely on other factors. Thus, more research is required to explore the efficacy of the new Nordic diet and its applicability to populations with different CVD risk factors.

In the present review, there were more studies included that investigated the MedD that any other regional diet. In all but one of the included studies, the MedD was shown to have significant positive effects on most outcomes of interest. Key features of the MedD typically include multiple daily servings of olive oil, tree nuts, fruits and vegetables and fresh fish, white meat and whole grains, while processed foods, grains, and red meat are advised to be consumed less than once daily (Figure 3) [54,85,86]. A plethora of meta-analyses have shown that adherence to the MedD reduces CV risk factors including hard endpoints [85,86]. Moreover, research also suggests that the MedD can be an effective remedy for precursors of CVD, including hypertension or obesity [87,88,89]. Nevertheless, great heterogeneity is observed with respect to the trials implementing MedD interventions, their design, fragility index, and intervention characteristics, as well as regarding what is considered as a MedD by definition [54].

The only study included that investigated the Mexican diet showed positive changes to Ins and reported no significant positive results for any other biochemical indices of CVD risk [35]. The nutritional information of the planned interventions showed that the Mexican diet and the US diet comparator contained approximately the same amount of calories, with participants prescribed a eucaloric diet depending on their own expenditure. The Mexican diet contained 32.3 g less sugar daily (18.6 g less fructose), which may explain why insulin concentrations were significantly reduced in the intervention arm.

Of the two included studies that investigated the TPM, one showed some significant positive improvements in the anthropometric and biochemical indices [33], and one showed none [34]. The nutritional intervention of Razmgah’s study [33] was poorly reported, and potential causal mechanisms cannot be explored. Hamidnia’s study [34] failed to reveal any significant positive changes between treatment arms. Thus, based on the available evidence, the effect of the TPM on CVD risk factors in patients at increased risk appears poor.

With regard to the Southern European Atlantic diet, the overall studies backing the “healthy diet” narrative are limited, and more research is required to identify the specific characteristics of this pattern and to associate its adherence with health outcomes.

### 4.3. Health Benefits of Regional Diets beyond CVD Risk

Apart from CVD, which was the main aim of the present systematic review, high quality research has revealed that regional dietary patterns may also be effective in other NCD categories. For instance, meta-analyses of RCTs reveal that the MedD can be an effective complementary therapy in patients with cancers [90], mental health problems [91], or diabetes [86,92,93] diagnoses. Similarly, the aggregation of RCTs implementing the new Nordic diet [94] also revealed improvements in the metabolic control of patients at risk for diabetes. With regard to the adoption of the traditional Chinese diet and medicine, meta-analyses of RCTs have showed selective improvements in patients with a cancer [95] or obesity diagnoses [96]. On the other hand, results from observational studies suggest that adherence to the traditional Japanese diet reduces the risk for developing specific cancers, and in parallel lower all-cause, CVD and cancer mortality [97,98].

### 4.4. Environmental Benefits of Regional Diets

In 1986, Gussow and Clancy [99] were the first to suggest the term “sustainable diet” to describe a diet composed of foods contributing to health and nutrient adequacy, while also contributing to the sustainability of food sources (agricultural system, or fishing). Thus, sustainable diets make use of human and natural resources to produce and consume foods “in a manner that is not wasteful of finite resources as topsoil, water, and fossil energy” [99]. Therefore, it appears that sustainability is multidimensional, with environmental, economic and social impacts, aiming to achieve multiple sustainable development goals (SDGs) in synergy. In this manner, changes in food availability, the industrialization of food systems, technological advances, globalization of the market and food policies and lobbies have all induced a nutrition transition deviating from the traditional regional diets. A return to the traditional regional patterns would not only improve population health, but also contribute to the sustainability of the environment, while taking into account the identity and diversity of food systems and cultures of each region [100].

### 4.5. Limitations of the Present Study

Limitations of the present systematic review include the relatively narrow spectrum traditional regional diets included in the analysis. It appears that several dietary patterns have not been examined yet, with respect to their effect on CVD risk. In the present search, no RCTs were found implementing other traditionally healthy diets with regard to CVD outcomes, including the Inuit (arctic) diet or the South Asian diet. Moreover, the present review also highlighted the great heterogeneity in the regional diets RCTs, not allowing for a synthesis of the evidence.

It should also be noted that an additional limitation of the studies included in the present systematic review involves the lack of biomarker use for assessing treatment adherence. A variety of biomarkers are available today and can confer greater validity when assessing dietary treatment adherence, as compared to the usual tools for dietary assessment. Some are food specific, evaluating fish, meat, or olive oil intake (including urine hydroxytysosol or serum γ-tocopherol concentrations) [101,102,103,104,105,106], whereas others are nutrient-specific. The use of such biomarkers might have resulted in different findings with regard to CVD endpoints.

Moreover, metabolic health often stems from the perfect balance of nutrients, with BP for example being affected by many micronutrients apart from sodium intake, including potassium, magnesium, calcium, fish taurine, or soy phytoestrogens. These interacting micronutrients were not assessed in the included studies and this might have reduced the efficacy of regional diets on participants’ BP health.

On a side note, in all of the research included herein, specific aspects of the traditional lifestyle beyond regional diets, including the siesta, reduced stress, improved quality of life, and social capital, cannot be transferred to an RCT design, although they might induce improved benefits [54].

Moreover, future research can also aggregate data from the RCTs implementing regional diets for the prevention of other NCD categories, in order to help us attain a better picture on the effects of traditional diets on NCD prevention.

## 5. Conclusions

Regional diets are sustainable dietary patterns that make use of locally sourced goods to nurture the surrounding populations. With regard to their CVD benefits, we cannot draw conclusions on whether one specific pattern is superior to another, although the majority of research involves the MedD. Selective CV outcomes (anthropometry, BP, inflammation markers) are improved following regional dietary interventions, although aggregation of data for the recommendation of specific territorial diets for the reduction of CVD risk is not yet feasible, with the possible exception of the MedD.

Although strengths and areas that benefit differ between the distinct target populations of the included RCTs, the reviewed body of evidence suggests that adherence to regional diets may reduce specific CV risk factors, with MedD appearing to offer a wider range of CV-related benefits. Nevertheless, the evidence is still limited for most of the examined regional diets and a great heterogeneity is observed. Thus, more research examining the effects of regional dietary interventions on CVD parameters is required to increase the body of existing evidence.

## Figures and Tables

**Table 1:** PICO of the study’s research question.

PICO Components	Determinants
Population:	Adults at high risk for CVD (age ≥ 55 years old and at least one of the following risk factors 1. post-menopausal, 2. with hyperglycemia or prediabetes, 3. hypertension, 4. hyperlipidemia, 5. overweight/obesity, 6. current cigarette smokers, or 7. excessive alcohol drinkers, 8. physically inactive)
Intervention:	Any regional diet, including the Mediterranean, Persian, Southern European Atlantic, Japanese, Chinese, Nordic, or other.
Comparison:	Habitual diet, sham diet, any other dietary pattern, or no intervention
Outcome:	Any CVD outcome, including BW, BMI, WC, body fat (% BW), waist-to-hip ratio, blood pressure, insulin resistance, fasting blood glucose/insulin, HbA1c, blood lipids, FMD or LDF

BMI, body mass index; BW, body weight; CVD, cardiovascular diseases; FMD, Flow Mediated Dilatation; HbA1c, glycosylated haemoglobin; LDF, laser Doppler Fluximetry; WC, waist circumference.

**Table 2:** Detailed search strings used for the PubMed database.

1	“Mediterranean diet”[Abstract] AND (“fatness”[Abstract] OR “body fat”[Abstract] OR “adiposity”[Abstract] OR “obesity”[Abstract] OR “obese”[Abstract] OR “BMI”[Abstract] OR “blood pressure”[Abstract] OR “glucose”[Abstract] OR “insulin”[Abstract] OR “cholesterol”[Abstract] OR “flow mediated dilation”[Abstract] OR “laser doppler”[Abstract] OR “weight”[Abstract] OR “circulation”[Abstract])
2	mexican diet[Abstract] AND (fatness[Abstract] OR body fat[Abstract] OR adiposity[Abstract] OR obesity[Abstract] OR obese[Abstract] OR BMI[Abstract] OR blood pressure[Abstract] OR glucose[Abstract] OR insulin[Abstract] OR cholesterol[Abstract] OR flow mediated dilation[Abstract] OR laser doppler[Abstract] OR weight[Abstract] OR circulation[Abstract])
3	persian diet[Abstract] AND (fatness[Abstract] OR body fat[Abstract] OR adiposity[Abstract] OR obesity[Abstract] OR obese[Abstract] OR BMI[Abstract] OR blood pressure[Abstract] OR glucose[Abstract] OR insulin[Abstract] OR cholesterol[Abstract] OR flow mediated dilation[Abstract] OR laser doppler[Abstract] OR weight[Abstract] OR circulation[Abstract])
4	chinese diet[Abstract] AND (fatness[Abstract] OR body fat[Abstract] OR adiposity[Abstract] OR obesity[Abstract] OR obese[Abstract] OR BMI[Abstract] OR blood pressure[Abstract] OR glucose[Abstract] OR insulin[Abstract] OR cholesterol[Abstract] OR flow mediated dilation[Abstract] OR laser doppler[Abstract] OR weight[Abstract] OR circulation[Abstract])
5	(“New Nordic diet”[Abstract] OR “Nordic diet”[Abstract]) AND (fatness[Abstract] OR “body fat”[Abstract] OR “adiposity”[Abstract] OR “obesity”[Abstract] OR “obese”[Abstract] OR “BMI”[Abstract] OR “blood pressure”[Abstract] OR “glucose”[Abstract] OR “insulin”[Abstract] OR “cholesterol”[Abstract] OR “flow mediated dilation”[Abstract] OR “laser doppler”[Abstract] OR “weight”[Abstract] OR “circulation”[Abstract])
6	“Japanese diet”[Abstract] AND (“fatness”[Abstract] OR “body fat”[Abstract] OR “adiposity”[Abstract] OR “obesity”[Abstract] OR “obese”[Abstract] OR “BMI”[Abstract] OR “blood pressure”[Abstract] OR “glucose”[Abstract] OR “insulin”[Abstract] OR “cholesterol”[Abstract] OR “flow mediated dilation”[Abstract] OR “laser doppler”[Abstract] OR “weight”[Abstract] OR “circulation”[Abstract])
7	“Atlantic diet”[Abstract] AND (“fatness”[Abstract] OR “body fat”[Abstract] OR “adiposity”[Abstract] OR “obesity”[Abstract] OR “obese”[Abstract] OR “BMI”[Abstract] OR “blood pressure”[Abstract] OR “glucose”[Abstract] OR “insulin”[Abstract] OR “cholesterol”[Abstract] OR “flow mediated dilation”[Abstract] OR “laser doppler”[Abstract] OR “weight”[Abstract] OR “circulation”[Abstract])

**Table 3:** Characteristics of the included RCTs applying regional diet interventions for the prevention of CVD.

Regional Diet	First author	Trial name	Design	Masking	Participant Characteristics	Intervention	Comparator(s)	Intervention Duration	Outcomes	Significant Findings Favoring Intervention
**Japanese**	Asano [27,28]	-	Parallel	Single-blind	*n* = 60 patients with overweight/obesity (20–70 years)	Older (circa 1975) Japanese diet (*n* = 30)	Modern Japanese diet (*n* = 30)	28 days	BW, BF mass, BMI, WC, SBP, DBP, TC, HDL, CRP, LDL, TG, ALT, ALP, γ-GT, AST, HbA1c, FPG, HOMA-IR, IRI, PLT, Hb, MCV, MCH, RBC, WBC, LD, UA, BUN, Cre, CK, TP, Fe, K, Na, Cl, Mg	BW, BF mass, BMI, WC, CRP, HDL, LDL, HbA1c
**Chinese**	Leonetti [29]	-	Parallel	NR	*n* = 284 patients with overweight/obesity (25–70 years)	Hypocaloric Chinese diet (1200 kcal) (*n* = 142)	Hypocaloric typical Western diet (1200 kcal) (*n* = 142)	6 weeks	BMI, LBM, hunger, PHI, MHI	BMI, LBM, hunger, PHI, MHI
**Nordic**	Adamsson [31]	NORDIET	Parallel	Open label	*n* = 86 patients with mild hypercholesterolemia (25–65 years)	Nordic diet (*n* = 44) (BMI: 26.3 kg/m^2^)	Usual Western diet (*n* = 42) (BMI: 26.5 kg/m^2^)	6 weeks	BW, BMI, SBP, DBP, Ins, Glu, HDL/LDL, TC, TG, LDL, HDL, apoB/apoA_1_, ApoB, ApoA_1_	BW, BMI, SBP, Ins, TC, LDL, HDL, LDL/HDL, apoB/apoA_1_, ApoB, ApoA_1_
	Poulsen [30]	-	Parallel	Open label	*n* = 181 men and women with abdominal obesity (18–65 years)	New Nordic diet based on 15 food groups (*n* = 113)	Average Danish diet (*n* = 68)	26 weeks	BW, BF mass, BF (% BW), BMI, WC, HC, SBP, DBP, TG, LDL, HDL, VLDL, FPG, Ins, HOMA-IR, Matsuda Index, CRP, fructosamine, Sagittal diameter, physical fitness	BW, BF mass, BF (% BW), BMI, WC, HC, SBP, HOMA-IR, Matsuda Index, TG, CRP, Saggital diameter
	Ulven [32]	-	Parallel	Double blind	*n* = 99 participants with elevated hs-CRP and TC (25–29 years)	Nordic diet with higher fiber and FA content (*n* = 47)	Typical modern Nordic diet (*n* = 52)	8 weeks	BW, TC, TG, LDL, HDL, FPG, Ins, HbA1c, SBP, DBP, hs-CRP, IL-6, ALT, IFNγ, sTNFR1, LpA, ApoB, ApoA_1_	BW, TC, TG, LDL, HDL
**MedD**	Bos [47]	-	Parallel	Open label	*n* = 39 non-diabetic, BMI ≥ 25 kg/m^2^ or WC ≥ 94 cm for men, ≥ 80 cm for women (40–65 years)	MedD (*n* = 19) (BMI: 26.1 kg/m^2^)	Western-style diet (*n* = 20) (BMI: 28.3 kg/m^2^)	10 weeks	BW, WC, TC, HDL, LDL, TG, TC:HDL, FPG, Ins	None
	Buscemi [46]	-	Parallel	Open label	*n* = 20 women with overweight/obesity (30–50 years) (BMI: 27–39.9 kg/m^2^)	Hypo-caloric MedD (*n* = 10) (BMI = 34 kg/m^2^)	Atkins (very low CHO) diet (*n* = 10) (BMI: 34.5 kg/m^2^)	2 months	FMD (%), TNF-α, Il-6, 8-iso-PGF2a, BW, BF (% BW), SBP, UA	BW, FMD
	Davis [48,49]	MedLey	Parallel	Open label	*n* = 149 older adults (≥65 years)	MedD (*n* = 80) (BMI: 26.7 kg/m^2^)	Usual diet (*n* = 69) (BMI: 27.1 kg/m^2^)	6 months	SBP, DBP, FMD (%), hs-CRP, LDL, HDL, TG, F2-IsoPs, Ins, FPG	SBP (3, 6 months), DBP (6 months), FMD, TG (3, 6 months), F2-IsoPs (3, 6 months)
	Duś-Żuchowska [36,37]		Parallel	Single blind	*n* = 131 post-menopausal women with obesity, at a risk of MetS	Hypo-caloric (−700 kcal/day) MedD (*n* = 68) (BMI: 33.8 kg/m^2^)	Energy-restricted Central European diet (*n* = 63) (BMI: 33.6 kg/m^2^)	16 weeks	hs-CRP, ADMA, BW, BF (mass), FFM, VAT, WC	BW, BF, FFM, VAT
	Jaacks [38]	-	Parallel	Open label	*n* = 30 patients with overweight/obesity (35 < BMI ≥ 28 kg/m^2^)	MedD (*n* = 11)	(1) Usual diet (*n* = 9) (2) Usual diet + fish oil, walnuts, and grape juice (*n* = 10)	8 weeks	BW, WC, FMD (%), Ins, TC, TG, LDL, HDL	BW, TC (at 4 weeks), LDL (at 4 weeks)
	Jennings [40]	-	Parallel	Open label	*n* = 1142 older adults (≥65 years)	MedD (*n* = 574) (BMI: 26.7 kg/m^2^)	Habitual diet (*n* = 568) (BMI: 26.6 kg/m^2^)	12 months	SBP, DBP	SBP
	Sofi [44]	CARDIVEG	Cross-over	Open label	*n* = 118 patients with BMI ≥ 25 kg/m^2^ and ≥1 of the following: TC > 190 mg/dL, LDL > 115 mg/dL, TG > 150 mg/dL, 110 ≤ FPG < 126 mg/dL (BMI: 30.6 kg/m^2^)	Hypo-caloric MedD (*n* = 118)	Hypo-caloric lacto-ovo vegetarian diet (*n* = 118)	3 months	BW, BMI, BF mass, TC, HDL, LDL, TG, FPG, Ins	LDL, TG
	Panizza [45]	Multiethnic Cohort Adiposity Phenotype	Parallel	Double blind	*n* = 60 patients with overweight/obesity (30–50 years) (BMI: 25–40 kg/m^2^)	IER MedD (*n* = 30) (BMI: 30.5 kg/m^2^)	Iso-energetic DASH diet (*n* = 30) (BMI: 30.8 kg/m^2^)	12 weeks	BW, BMI, WC, BF mass, BF (% BW), TC, HDL, LDL, TG, SBP, DBP, VAT, ALT	BW, VAT, ALT, BMI, WC, BF mass, BF (% BW)
	Wade [42]	MedPork	Cross-over (8 wk interval)	Open label	*n* = 33 patients at risk for CVD (SBP > 120 mmHg and ≥2 of the following: BMI ≥ 25 kg/m^2^; dyslipidemia (TC ≥5.5 mmol/L, TG > 2.0 mmol/L, LDL ≥ 3.5 mmol/L, HDL ≤ 0.9 mmol/L for men or ≤1.0 mmol/L for women); IFG (6.1–7.8 mmol/L); family history of CVD/T2DM) (BMI: 30.6 kg/m^2^)	MedD supplemented with 2–3 serv/wk of fresh, lean pork (*n* = 33)	Low-fat diet (*n* = 33)	8 weeks	BW, BMI, CRP, WC, BF mass, BF (% BW), TC, HDL, LDL, TG, SBP, DBP, FPG, Ins	BW, BMI, WC
	Wade [43]	MedDairy	Cross-over (8 wk wash-out)	Open label	*n* = 41 patients with hypertension and ≥2 of the following: overweight, IFG, dyslipi-demia, family history of CVD/T2DM (BMI: 30.8 kg/m^2^)	MedD with 3–4 serv of dairy/day (*n* = 41)	Low-fat diet (*n* = 41)	8 weeks	BW, BMI, WC, BF mass, BF %, TC, HDL, LDL, TG, SBP, DBP, FPG, Ins	SBP, TG, HDL, BW, BF mass, BF (% BW)
	Wardle [39]	-	Parallel	Single blind	*n* = 155 patients with mildly/moderately elevated TC (adults)	MedD (*n* = 53)	(1) Usual diet (*n* = 50) (2) Low-fat diet (*n* = 52)	12 weeks	BW, TC, LDL, HDL, TG, stress, cognitive function, well-being, depression, etc.	TC, LDL, BW
**Mexican**	Santiago-Torres [35]	-	Cross-over (28 d wash-out)	Single blind	*n* = 26 women with overweight/obesity (BMI: 30 kg/m^2^)	Traditional MexD (pre-1940s), based on data from historical records (*n* = 26)	US diet (*n* = 26)	24 d (each arm)	FPG, Ins, CRP, IGF-1, IGFBP-3, adiponectin, IL-6, HOMA-IR	Ins
**Persian**	Ghayour Razmgah [33]		Parallel	Open label	*n* = 43 patients with NAFLD (grades 1–2), diagnosed by US imaging (20–60 years)	TPM diet (*n* = 21) (BMI: 26 kg/m^2^)	Low-fat hypo-caloric diet (*n* = 22) (BMI: 24.4 kg/m^2^)	12 weeks	BMI, ALT, AST, changes in NAFLD grade	BMI, NAFLD grade, AST (6 weeks), ALT (6 weeks)
	Hamidnia [34]		Parallel	Single blind	*n* = 69 women with overweight (BMI: 27–29.9 kg/m^2^, WC > 88 cm)	Hypocaloric TPM diet (1200–1600 kcal/day) (*n* = 23) (BMI: 29.1 kg/m^2^)	(1) Hypo-caloric diet (1200–1600 kcal/day) + orlistat (120 mg/day) (*n* = 23) (BMI: 29.4 kg/m^2^) (2) Hypo-caloric diet (1200–1600 kcal/day) + MDB ONS (2 × 5 g/day), (*n* = 23) (BMI: 28.5 kg/m^2^)	12 weeks	BW, BMI, WC, BF (mass and % BW), TC, HDL, LDL, TG, Ins	None
**Atlantic**	Calvo-Malvar [24]	GALIAT	Parallel	Open label	*n* = 250 families (720 adults and children)	Educational sessions, cooking classes, written supporting material, and foods that form part of the Atlantic diet	Habitual lifestyle	6 months	BW, BMI, BF, HWR, ΤC, HDL, LDL-C, CRP, TNF-α, FPG, HOMA-IR, SBP, DBP	BW, BF, BMI, HWR, TC, LDL

8-iso-PGF2a, 8-iso-prostaglandin F2a; ADMA, asymmetrical dimethylarginine; ALB, albumin; ALT, alanine transaminase; ALP, alkaline phosphatase; apoA1, apolipoprotein A1; apoB, apolipoprotein B; AST, aspartate aminotransferase; BF, body fat; BMI, body mass index; BUN, blood urea nitrogen; BW, body weight; Ca, Calcium; CK, creatine kinase; CARDIVEG, Cardiovascular Prevention With Vegetarian Diet; Cl, cloride; Cre, creatinine; CRP, c-reactive protein; CVD, cardiovascular disease; DASH, dietary approach to stop hypertension; DBP, diastolic blood pressure; F2-IsoPs, F2-isoprostanes; FA, fatty acids; Fe, Iron; FFM, fat-free mass, FMD, flow-mediated dilation; FPG, fasting plasma glucose; GALIAT, Galicia Alimentación Atlántica; Hb, hemoglobin; HbA1c, glycosylated haemoglobin; HC, hips circumference; HDL, high density lipoprotein; HOMA-IR, insulin resistance homeostatic model of assessment; hs-CRP, high sensitive C-reactive protein; HWR, hip-to-waist ratio; IDF, International Diabetes Federation; IER, intermittent energy restriction; IGF-1, insulin growth factor 1; IGT, impaired glucose tolerance; IGFBP-3, insulin-like growth factor binding protein 3; IL-6, interleukin 6; INFγ, interferon γ; Ins, insulin; IRI, immune-reactive insulin; K, potassium; LD, lactate dehydrogenase; LDL, low density lipoprotein; LpA, lipoprotein A; MCH, mean corpuscular Hb; MCV, mean corpuscular volume; MedD, Mediterranean diet; MexD, Mexican diet; MDB, Majoon Davaye Balgham; MedLey, Mediterranean diet for cognitive and cardiovascular health in the elderly; MetS, Metabolic Syndrome; Mg, magnesium; MHI, mental health index; Na, sodium; NAFLD, non-alcoholic fatty-liver disease; NCEP-ATP III, National Cholesterol Education Program—Adult Treatment Panel III; NR, not reported; OHA, oral hypoglycemic agents; ONS, oral nutrient supplements; PHI, physical health index; PTL, platelets; RBC, red blood cells; RDN, registered dietitian-nutritionist; SBP, systolic blood pressure; SEAD, Southern European Atlantic diet; serv, serving; sTNFR1, soluble tumor necrosis factor receptor 1; T2DM, type 2 diabetes mellitus; TC, total cholesterol; TG, triglycerides; TMAO, trimethylamine N-oxide; TNF-a, tumor-necrosis factor a; TP, total protein; TPM, Traditional Persian Medicine; UA, uric acid; BUN, blood urea nitrogen; US, ultrasound; VAT, visceral adipose tissue; *VLDL*, very low density lipoprotein; *WBC*, white blood cells; *WC*, waist circumference; ^†^ mean standard deviation.

**Table 4:** Overview of CVD-specific outcomes (favoring the intervention arm) in RCTs implementing regional dietary patterns among participants with increased CVD risk.

Regional Dietary Pattern	Reference	Anthropometric Indices							Inflammation Markers						Blood Lipid Concentrations							Blood Pressure		FMD (%)	ADMA (nmol/mL)	Glucose Metabolism			
		BW (kg)	BMI (kg/m^2^)	BF (% BW or kg)	VAT (cm^2^)	WC (cm)	FFM/LBM (% BW or kg)	HWR	CRP (mg/L)	hs-CRP (mg/L)	TNF-α (pg/mL)	sTNFR1 (pg/mL)	IL-6 (pg/mL)	IFNγ (pg/mL)	TC (mg/dL)	HDL (mg/dL)	LDL (mg/dL)	TG (mg/dL)	apoA_1_ (g/L)	apoB (g/L)	apoB/apoA_1_	SBP (mm Hg)	DBP (mm Hg)			Ins (mg/dL)	HbA1c (%)	FPG (mg/dL)	IR *
Japanese	Asano [27,28]	↓	↓	↓		↓	-		↓						↓	↑	↓	-				-	-				↓	-	-
Chinese	Leonetti [29]		↓				↓																						
Nordic	Adamsson [31]								-						↓	↓	↓	-	↓	↓	↓	↓	-			↓		-	↓
	Poulsen [30]	↓		↓		↓	↓		↓						-	-	-	↓				↓	↓			-		-	-
	Ulven [32]	↓								-		-	-	-	↓	↓	↓	↓	-	↓		-	-			-	-	-	
MedD	Bos [47]	-				-									-	-	-	-								-		-	
	Buscemi [46]	↓		-							-		-									-		↑					
	Davis [48,49]									-						-	-	↓				↓	↓	↑		-		-	
	Duś-Żuchowska [36,37]	↓		↓	↓	-	↓			-															↓				
	Jaacks [38]	↓				-									↓	-	↓	-						-		-			
	Jennings [40]																					↓	-						
	Sofi [44]	-	-	-											-	-	↓	↓								-		-	
	Panizza [45]	↓	↓	↓	↓	↓									-	-	-	-				-	-						
	Wade [42]	↓	↓	-		↓			-						-	-	-	-				-	-			-		-	
	Wade [43]	↓	-	↓		-									-	↓	-	↓				↓	-			-		-	
	Wardle [39]	↓													↓	-	↓	-											
Mexican	Santiago-Torres [35]								-				-													↓		-	-
Persian	Ghayour Razmgah [33]		↓																										
	Hamidnia [34]	-	-	-		-									-	-	-	-								-			
Atlantic	Calvo-Malvar [24]	↓	↓	↓				↓	-		-				↓	-	↓	-				-	-					-	-

ADMA, asymmetric dimethylarginine; apoA_1_, apolipoprotein A_1_; ApoB, apolipoprotein B; BMI, body mass index; BF, body fat; BW, body weight; CRP, c-reactive protein; DBP, diastolic blood pressure; FFM, fat-free mass; FMD, brachial artery flow-mediated dilation; FPG, fasting blood glucose; HbA1c, glycosylated haemoglobin; HDL, high density lipoprotein; HOMA-IR, insulin resistance homeostatic model of assessment; hs-CRP, high-sensitivity CRP; HWR, hips-to-waist ratio; IL-6, interleukine 6; IFNγ, interferon-γ; Ins, insulin; IR, insulin resistance; LBM, lean body mass; LDL, low density lipoprotein; MedD, Mediterranean diet; SBP, systolic blood pressure; TC, total cholesterol; TG, triglycerides; TNF-α, tumor-necrosis factor α; sTNFR1, soluble TNF receptor 1; VAT, visceral adipose tissue; WC, waist circumference; * Based on the HOMA-IR or the Matsuda Index; ↓ reduced; ↑ elevated.

## Data Availability

All data are presented within the manuscript text.
